# Draft genome sequences of *Listeria monocytogenes* and *Listeria innocua* isolated from frozen corn in Japan

**DOI:** 10.1128/mra.00183-25

**Published:** 2025-04-29

**Authors:** Shiori Yamamoto, Yumiko Okada

**Affiliations:** 1 Department of Nutrition and Dietetics, Kamakura Women’s University, Kamakura, Kanagawa, Japan; 2 Division of Biomedical Food Research, National Institute of Health Sciences, Tonomachi, Kawasaki, Kanagawa, Japan; Queens College Department of Biology, Queens, New York, USA

**Keywords:** *Listeria monocytogenes*, *Listeria innocua*, frozen corn, genome sequences

## Abstract

Six *Listeria* spp. strains were isolated from frozen corn in Japan. Here, we report the draft genome sequences of these strains. The genome sizes ranged from 2,853,850 to 3,062,263 bp at 381× to 564× coverage.

## ANNOUNCEMENT

*Listeria* is a genus of gram-positive, short rod-shaped bacteria that can grow at refrigerating temperatures and includes 28 species (1). Among these species, *Listeria monocytogenes* is a foodborne pathogen that causes listeriosis, leading to meningitis and miscarriages, and is isolated from ready-to-eat foods such as natural cheeses, smoked salmon, and raw ham. Conversely, other species, such as *Listeria innocua*, have been investigated as indicator bacteria for foods in food facilities because they are widely present in natural environments (2). It has been reported that frozen vegetables have the potential to cause listeriosis outbreaks (3, 4); however, the prevalence and genomic information concerning *Listeria* spp. in frozen produce remain limited. Here, we sequenced *Listeria* spp. isolates from frozen corn to characterize these genetic traits. The *Listeria* spp. strains were isolated from retail frozen corn in Japan; briefly, 25 g of each sample was homogenized with 225 mL of demi-Fraser broth (Neogen) and enriched at 37°C for 24 h and performed the assay using the 3M Molecular Detection Assay 2*—Listeria monocytogenes* (Neogen) to identify *L. monocytogenes*-positive cultures. The positive culture was spread onto CHROMagar *Listeria* (Chromagar) and incubated at 37°C for 48 h, after which typical colonies were isolated.

Six *Listeria* spp. isolates (20V-2-1, 20V-2-6, 20V-6-1, 20V-6-4, 20V-6-5, and 20V-7-1), from different corn samples, were incubated on brain heart infusion agar at 37°C for 18 h, after which the genomic DNA of the isolates was extracted from a single colony using a Maxwell RSC blood DNA kit (Promega). The DNA library (approximately 200–300 size) was prepared from 1 µg DNA using the NEBNext Fast DNA Fragmentation and Library Prep set (New England Biolabs) and was analyzed using an Ion Chef/GeneStudio S5 system (Life Technologies). Quality trimming and *de novo* assembly of the raw reads were performed using CLC Genomics Workbench version 24.0 (Qiagen), and the obtained contigs were annotated using DDBJ Fast Annotation and Submission Tool (DFAST) version 1.6.0 (5). All analyses used default parameters for the software.

**Fig 1:**
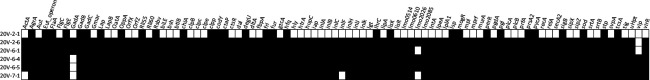
Virulence factors present or absent in the six *Listeria* spp. strains. Black boxes indicate the presence of the factor, and white boxes indicate its absence.

The number of raw reads obtained from the six strains ranged from 4,967,663 to 7,658,227 and were assembled into 42–89 contigs with accumulated lengths of 2,853,850–3,062,263 bp at 381× to 564× coverage (Table 1). The sequences included 37.3%–37.9% G + C content and 2,846–3,056 coding sequences. The 20V-2-1 strain was identified as *L. innocua* belonging to CC1008/ST1008, and the other five strains were *L. monocytogenes* belonging to the lineage II type and various sequence types using the Institut Pasteur MLST website scheme (6). A total of 30 genes associated with virulence factors were identified in the *L. innocua* isolate, and 87–90 genes were identified in the *L. monocytogenes* isolates by *in silico* analysis using VirulenceFinder 2.0 (7) (Fig. 1). These results provide genome sequence data for *L. monocytogenes* and *L. innocua* strains isolated from frozen corn and suggest that these strains may pose potential risks to human health. Furthermore, additional data are needed to understand *Listeria* spp. in foods.

**TABLE 1:** Genome sequences of six *Listeria* spp. strains

Parameter	Strains					
	20V-2-1	20V-2-6	20V-6-1	20V-6-4	20V-6-5	20V-7-1
Genome size (bp)	2,987,150	3,062,263	2,999,782	2,853,850	2,868,389	3,018,180
Coverage depth	437	564	511	381	425	384
No. of Contigs	86	56	89	42	48	43
GC Content (%)	37.3	37.8	37.8	37.9	37.9	37.8
N50	54,984	102,859	61,327	103,635	94,380	131,748
Coding sequences	2,991	3,056	2,983	2,846	2,854	3,004
rRNA	3	2	4	3	3	2
tRNA	53	55	44	55	50	48
CRISPRs	1	0	1	2	2	5
BioSample accession	SAMD00805288	SAMD00805289	SAMD00805290	SAMD00805291	SAMD00805292	SAMD00805293
SRA accession	DRR596087	DRR596088	DRR596089	DRR596090	DRR596091	DRR596092
GenBank accession	BAAFVD010000000	BAAFVE010000000	BAAFVF010000000	BAAFVG010000000	BAAFVH010000000	BAAFVI010000000
Species	*L. innocua*	*L. monocytogenes*	*L. monocytogenes*	*L. monocytogenes*	*L. monocytogenes*	*L. monocytogenes*
Lineage^*a*^	−	II	II	II	II	II
MLST	CC1008/ST1008	CC204/ST204	CC8/ST8	CC19/ST398	CC19/ST398	CC121/ST121
Virulence genes	30	90	89	90	90	87

^*a*^ Lineage for L. monocytogenes.

## Data Availability

Draft genome sequences have been deposited in DDBJ/ENA/GenBank under the accession numbers provided in Table 1. The raw sequence data have been deposited in the DRA under the BioProject accession number PRJDB18585.
